# The cortisol awakening response in a 3 month clinical trial of service dogs for veterans with posttraumatic stress disorder

**DOI:** 10.1038/s41598-023-50626-y

**Published:** 2024-01-18

**Authors:** Leanne O. Nieforth, Kerri E. Rodriguez, Run Zhuang, Elise A. Miller, Arman Sabbaghi, A. J. Schwichtenberg, Douglas A. Granger, Marguerite E. O’Haire

**Affiliations:** 1 Comparative Pathobiology, Center for the Human-Animal Bond, Purdue University, West Lafayette, United States; 2 College of Veterinary Medicine, University of Arizona, Oro Valley, AZ USA; 3 Unlearn.AI, Inc., San Francisco, USA; 4 Human Development and Family Science, Purdue University, West Lafayette, USA; 5 Institute for Interdisciplinary Salivary Bioscience Research, University of California at Irvine, Irvine, USA; 6 Department of Pediatrics, Johns Hopkins University School of Medicine, Baltimore, USA

**Keywords:** Human behaviour, Endocrinology

## Abstract

Recent literature suggests that service dogs may be a valuable complementary intervention option for posttraumatic stress disorder (PTSD) among military veterans due to the potential influence on stress response dysregulation. The aim of this short-term longitudinal study was to quantify the impact of service dogs in US military veterans with PTSD with particular attention to the cortisol awakening response. A sub aim of the study was to empirically evaluate the physiological effects of PTSD service dogs on veteran partners. We conducted a clinical trial (ID: NCT03245814) that assessed the cortisol awakening response for 245 participants at baseline and 3 months follow-up across an intervention group (service dog: veterans *n* = 88, partners *n* = 46) and control group (usual care: *n* = 73, partners *n* = 38). A total of *N* = 161 veterans and *N* = 84 partners collected whole saliva samples via a passive drool collection immediately upon waking, 30 min after waking, and 45 min after waking on three consecutive weekdays at baseline and again at follow-up. Mixed model repeated measures (MMRM) with a fixed effect of the intervention group (service dog or control) were utilized. Covariates considered for the model included time of awakening, sleep duration, sleep efficiency, prior day experiences (measured via ecological momentary assessment), traumatic brain injury, age, gender, race, ethnicity, socioeconomic status, smoking status, alcohol use, physical health, and body mass index. A total of 3951 salivary samples were collected (veterans: 2613, partners: 1338). MMRM results demonstrated that veterans with a service dog had a statistically significant higher cortisol awakening response, including the area under the curve with respect to both increase (AUCi, β = 1.46, *p* = 0.046) and absolute increase (AINC, β = 0.05, *p* = 0.035). Results were not statistically significant for partners. Findings suggest that veterans with service dogs have a higher, less blunted CAR in comparison to veterans receiving usual care alone. In veterans with a blunted morning cortisol response, service dog placement could help boost their morning cortisol response.

## Introduction

Service dogs have been investigated as a valuable complementary intervention option for posttraumatic stress disorder (PTSD) among some military veterans^1^. Though the literature on the benefits and challenges of service dogs for veterans with PTSD is growing, few studies explore the effects of the service dog on veterans from a physiological perspective. The current study empirically identifies the impact that service dogs may have on the stress physiology of veterans and veteran partners through analysis of the cortisol awakening response (CAR).

### Service dogs for veterans with PTSD

Multiple studies using standardized clinical measures suggest that PTSD service dogs may be associated with lower PTSD severity, lower depression, and higher quality of life^1,2^. In addition, qualitative studies report improvements in personal wellbeing and relationship health^3,4^. Alongside positive findings, drawbacks such as relationship challenges and difficulties with access and acceptance in public spaces have also been found^3,5–7^.

Only three studies to date have incorporated physiological data to explore the effects of service dogs for veterans with PTSD. One longitudinal study looked at changes in physical activity and sleep after a veteran was paired with a service dog using actigraphy^8^. This study found that pairing a veteran with a PTSD service dog improved both physical activity and sleep quality. Two other cross-sectional studies examined the stress hormone cortisol^9,10^. The first study focused only on the CAR (0 and 30 min after waking) and found that veterans with service dogs had a higher CAR than veterans on the waitlist for a service dog^9^. The second study incorporated both CAR samples (0, 15, 30, and 60 min after waking) and an evening sample (right before going to sleep) and found no difference between groups^10^. More studies incorporating physiological metrics are needed to further understand the biological processes and potential mechanisms that may play a role in the service dog intervention. These mechanisms are important to uncover as historically, human-animal interaction literature has focused predominantly on psychological processes rather than biological processes^11^. The current analysis adds to the literature by exploring the CAR as a potential biological process affected by the interaction of veterans and service dogs in the context of a 3 month clinical trial.

### PTSD service dogs and veteran families

The potential influence of a service dog on the family beyond the veteran (with whom the dog is matched), is an emerging area of research. Benefits of the service dog for the family include acting as a “relational bridge,” building resilience, and helping the veteran be more involved in family activities^3,6,12^. Alongside these family-focused benefits, challenges have emerged, including increased caregiver burden, decreased caregiver satisfaction, and jealousy^7,13,14^. Thus far, findings suggest that the benefits outweigh the challenges in most cases. The current literature on PTSD service dogs and veteran families has yet to use a physiological measure to explore potential family influences. This manuscript seeks to fill this gap in the current literature by measuring the CAR in both veterans and veterans’ partners.

### Cortisol awakening response

The hypothalamic-pituitary-adrenocortical (HPA) axis is a cascade of endocrine pathways that help the body to maintain homeostasis when challenged by stressors^15,16^. Dysregulation of the HPA axis has been implicated in the relationship of chronic stress and negative health effects^17,18^. The hormone cortisol, commonly explored as a biomarker of stress, is a product of the HPA axis^19^. The HPA axis follows a diurnal pattern of secretion with a peak level of cortisol occurring in the early morning^15^. This early morning peak is an indicator of the dynamic changes that occur from the cortisol awakening response (CAR).

There is an increasing amount of evidence to suggest that PTSD is associated with a dysregulation of the HPA axis. Specifically, it is proposed that prolonged hyperarousal-induced cortisol production can have adverse effects on the body’s ability to regain homeostasis and result in persistent alterations in HPA axis functioning^20,21^. Therefore, while individuals with exposure to chronic stress or trauma initially exhibit higher cortisol levels, repeated disruptions to the HPA axis feedback mechanism can result in blunted cortisol production and lower CARs^22–24^. Indeed, a number of studies and meta-analyses suggest that individuals with PTSD have a significantly reduced CAR and daily cortisol output in comparison to trauma-exposed controls without PTSD^25,26^. However, regulation of the HPA axis and its response to traumatic events is complex and variable among individuals^27^. Some studies suggest that CAR curves are less sensitive and flatter in individuals with PTSD^24,25,28,29^, while others suggest no connection between PTSD status and CAR^30–33^. These mixed findings reflect complexity regarding the role of genetics, pre-traumatic risk factors, and aspects of PTSD pathology and measurement^34,35^. Despite mixed findings, the CAR is one of the most non-invasive, available metrics to study the stress response system in the context of trauma.

### Theoretical basis

A prominent theory in the field of human-animal interaction is attachment theory, which suggests that humans are biologically motivated to form bonds with caregiving figures, creating a secure base throughout their lives to satisfy basic needs of safety^36^. The attachment system, as a mechanism for survival, turns on when an individual experiences stress^37^. Throughout life, an individual develops working models of attachment in terms of responsiveness to others’ bids for interactions and the ability to achieve sufficient attachment for oneself^38^. These models are critical in how individuals experience stress throughout their lives. The human-animal bond can provide the features of an attachment relationship in a similar way to a human–human bond^39^. Service dogs may influence these working models of attachment, potentially affecting stress levels^40^. This analysis adds another lens to understanding this potential theoretical mechanism underlying the interaction between veterans and service dogs by measuring the CAR as a physiological biomarker for stress.

The aim of this study was to empirically evaluate the physiological effects of service dogs on military veterans with PTSD through analysis of the CAR. The sub aim of the study was to investigate the physiological effects of PTSD service dogs on veteran partners. To address these aims, we conducted a clinical trial assessing the CAR at baseline and at 3 months follow-up across an intervention group (service dog) and control group (waitlisted with unrestricted access to usual care).

## Methods

### Participants

Participants were recruited from a United States, non-profit service dog provider, K9s For Warriors. A total of 245 individuals collected whole saliva samples, including 161 veterans and 84 partners. This sample is completely independent of the previous Rodriguez et al., 2018 study^9^. A power analysis (*d* = 0.40, power = 0.80, alpha = 0.05) was conducted on the primary outcome of the clinical trial, veteran PTSD severity, and suggested that the required number of participants was 50 per group (N = 100 veterans). Inclusion criteria for veterans consisted of a) a PTSD diagnosis verified by an independent clinician through the Clinician-Administered PTSD Scale, and b) an approved application for a service dog from K9s For Warriors. K9s for Warriors required the following criteria to be met for a veteran to receive a service dog: (a) military service on or after 9/11/2001, (b) honorable discharge or current honorable service, (c) a diagnosis of PTSD, traumatic brain injury or military sexual trauma from a medical professional, and (d) no conviction of any crimes against animals. Partners were invited to participate if they were cohabitating with a participating veteran.

### Procedure

This analysis was part of a preregistered clinical trial (clinicaltrials.gov ID NCT03245814, 10/08/2017) which was approved by the Purdue University Human Research Protection Program Institutional Review Board (IRB Protocol 1702018766) and the Purdue University Institutional Animal Care and Use Committee (IACUC Protocol 1702001541). The trial began in July 2017 and was completed in June 2020. The study was monitored by an independent Data and Safety Monitoring Board (DSMB) and all methods were carried out in accordance with relevant guidelines. Informed consent was obtained from all study participants. Due to the long existing waitlists for service dogs, complete randomization was not possible. To account for time on the waitlist and order of applications, veterans were block randomized (block size = 4, time difference between waitlist placement or service dog allocation = 1–3 months). Participants were then placed into the service dog group if their randomized schedule included receiving a dog directly after baseline while participants were placed in the control group if their randomized schedule dictated that they would not receive a service dog during the 3 month study period.

Service dogs from K9s For Warriors were predominantly sourced from shelters after a temperament screening and ensuring they were of appropriate size (24 inches at the shoulder). Service dogs received a minimum of 120 h of training by K9s For Warriors trainers before placement, ensuring that the dogs knew both basic tasks and tasks specific to mitigate PTSD symptoms. The five key PTSD specific tasks trained by the provider included: alert to anxiety, comfort from anxiety, cover (standing behind the veteran to notify of people approaching from behind), make a friend (social initiation) and block (making space between a veteran and another person)^41^. After this initial training, veterans and service dogs were paired during a two-week intensive training camp where K9s For Warriors staff trained the veterans as to how to interact and work with their dogs. There were no significant changes made to service dog training or placement strategies by the organization for the duration of the study.

The clinical trial included a blinded clinician assessment, standardized self-report clinical assessments, ecological momentary assessment, actigraphy, and saliva collection at baseline and 3 months after baseline (follow up). The focus of the current manuscript was to analyze the salivary cortisol data of veterans. A sub focus of the manuscript was to analyze the salivary cortisol data of the veterans’ partners. Other data streams are published elsewhere^6,13,14,42,43^.

### Salivary cortisol sampling protocol

Veterans and partners collected whole saliva via passive drool samples on three consecutive typical weekdays at baseline and again at follow up. Samples were requested to be collected immediately upon waking, 30 min after waking, and 45 min after waking following recommendations within the literature^30^. Participants were advised to refrain from eating, brushing their teeth, smoking, or drinking anything aside from water until the collection ended for the day^44^. Participants were told that they could take any necessary medication with water during the collection period.

Participants were sent reminder text messages (Zipwhip, 2017) to aid in collection compliance. Research assistants programmed the software to send reminders on collection days at times that were based upon the wake-up times that participants had previously shared with the research team. Upon receiving the reminders, participants were asked to obtain the samples and then reply to the message as a compliance marker and timestamp for each sample.

After collecting all three samples on a collection day, participants were instructed to keep the samples in their freezer until shipping all nine samples back to the research team after the final collection day. Samples were shipped overnight via pre-paid shipping envelopes. Upon arrival at the lab, the samples were kept frozen at − 80 °C until shipped for assay where they were kept at − 20 °C. Samples were assayed using a high sensitivity enzyme immunoassay at the Salimetrics’ Saliva Lab (Carlsbad, CA) using the Salimetrics Salivary Cortisol Assay Kit (Cat. No. 1-3002). The assay had an average intra-assay coefficient of variation of 4.60% and an average inter-assay coefficient of variation 6.00%.

### Measures

#### Cortisol awakening response (CAR)

A CAR curve was created for each day using the three time points (upon waking (s1), 30 min after waking (s2), and 45 min after waking (s3)), totaling 3 CAR curves per participant over 3 days of collection. CAR was calculated as the “absolute increase of cortisol (AINC = (max value of s2, s3) − s1)” as used in previous literature focused on measuring CAR in a military population^45,46^. In addition to CAR, the area under the curve with respect to increase (AUCi) was calculated to measure cortisol awakening response over time^30^. AUCi (area under curve with respect to increase) was calculated with Pruessner’s formulas^47^.

#### Actigraphy

Sleep and activity were measured with the use of an Actiwatch Spectrum Plus (Philips Respironics). Actigraphy data was scored consistent with gold standard procedures, considering ecological momentary assessment morning sleep diary responses. All actigraphy scorers (n = 8) achieved 80% reliability and difficult cases were discussed as a research team. Actigraphy variables included rise time, sleep duration (the length of time the participant was asleep during their relative nighttime as defined by their own self-reported typical sleep–wake cycle) and sleep efficiency (percentage of time asleep between sleep onset and offset). Minute-by-minute (epoch-by-epoch) actigraphy was used to calculate the total number of minutes the participant was active in the 60 min prior to their time of awakening as an indication of participant compliance.

#### Ecological momentary assessment (EMA)

Participants filled out one EMA questionnaire in the morning, two at random times during the day, and one questionnaire before they went to bed. The morning survey was sent at the participant’s wakeup time. The random surveys were constrained to a window starting 2 h after wakeup until 2 h before bedtime separated by a minimum of 4 h. Expected wakeup and bedtimes were shared by participants prior to the study period start. This study uses data from the morning survey (if veteran had any nightmares the night before or panic attacks the day before) and the daily surveys (positive and negative affect). Nightmares were included as a binary yes/no variable, while panic attacks were captured both as a binary yes/no variable and as the number of panic attacks the participant experienced since the evening check-in the day prior. Affect was included in the model as total positive affect score minus total negative affect score, where higher scores indicate more positive affect (range: − 30 to 30)^48^. Affect was measured using a modified version of the Discrete Emotions Questionnaire and the Positive and Negative Affect Scale^49,50^.

#### Traumatic brain injury (TBI)

Screening for a TBI was conducted via the self-report Brief Traumatic Brain Injury Scale^51^, a three-question preliminary questionnaire for determining if a military member may be at risk for a deployment-related TBI.

#### Alcohol use

The Patient-Reported Outcomes Measurement Information System (PROMIS) Alcohol Use- Short Form 7a was used in the study to determine self-reported alcohol use of participants^52^. The survey is eight questions with the first question determining if the individual uses alcohol and the following seven questions determining the type and extent of use, if applicable.

#### Physical health

The Veterans’ Rand Survey (VR-12) Physical Health component score was used to measure self-reported physical health of participants^53^. This subscale focuses on physical abilities, physical limitations, and associated pain with physical exertion.

#### Demographics

Demographics included age, gender, race/ethnicity (aggregated into a binary score of Black, Indigenous, and Person of Color or White), socioeconomic status (comfortable, just enough to make ends meet, not enough to make ends meet), smoking status (binary yes/no), body mass index. Body mass index was calculated with patient reported weight and height (weight in kg/(height in meters)^2^).

### Data analysis

Given that the peak CAR occurs between 30 and 45 min after awakening^54^, accurate sampling is critical because inaccurate sampling would occur outside of the response pattern of interest. Non-adherence was defined as the participant not complying with the time stamps (0, 30, 45 min) or not following the sampling protocol (e.g., eating or smoking). When these instances occurred, the 3 days sampling protocol was rescheduled, and new supplies were sent to the participant. Following data collection, suspected non-adherence was further addressed using protocols from prior literature by which values where s2 < s1 were removed^55,56^. Multiple imputation by chained equations (MICE) was used to impute dropped and/or missing values as well as missing covariate data using SAS 9.4. Data was winsorized (*n* = 9 veterans, *n* = 2 partners) based on +/− 3 standard deviations from the mean^9,57^. Participants were excluded if they were pregnant (*n* = 1 veteran, *n* = 2 partners) or if they were on glucocorticoid medications (*n* = 7 veterans, *n* = 1 partner). We assumed that the missingness is missing at random such that the missingness can be fully explained by our other covariates. Due to the large variability in our measurements (and our imputation model), a total of 100 datasets were imputed.

Analyses were implemented in SAS 9.4 using the PROC MIXED procedure to fit a mixed model repeated measure (MMRM). The veteran model had a fixed effect of the intervention group (service dog or usual care). Covariates considered for the model included (as fixed effects): actigraphy variables (morning rise time, sleep duration, sleep efficiency, pre-awakening activity (number of minutes active in the last 60 min of sleep)); prior day experiences as measured via EMA (nightmares, panic attacks, positive and negative affect); and self-reported demographic variables (traumatic brain injury, age, gender, race/ethnicity, socioeconomic status, smoking status, alcohol use, physical health, and body mass index)^44^. Covariates were included in the final model based on model fit. The included variables in all models were the baseline score (e.g., mean baseline AUCi for the 3 days for the AUCi model), positive and negative affect score, physical health, number of panic attacks, and pre-awakening activity. Variable selection was conducted using backward selection with a stopping threshold of *p* < 0.3. Pre-awakening activity was considered to be an important covariate and therefore not used in the backward selection procedure. There were no random effects, and the error matrix was modeled over time using a banded main diagonal structure which contains no correlation between timepoints. This covariance structure was selected based on AIC and BIC criterion that was compared against the unstructured covariance matrix, which estimated weak correlations between the timepoints. Diagnostic graphics such as the Q–Q plot of residuals were used to confirm the normality assumption for residuals. For each imputed dataset, the MMRM was fit. Rubin’s rule was used to account for the variability between datasets and conduct inference on parameter estimates. Analysis of the partner data followed a similar procedure, though the percent missingness was lower and therefore imputation was not conducted.

## Results

**Figure 1:**
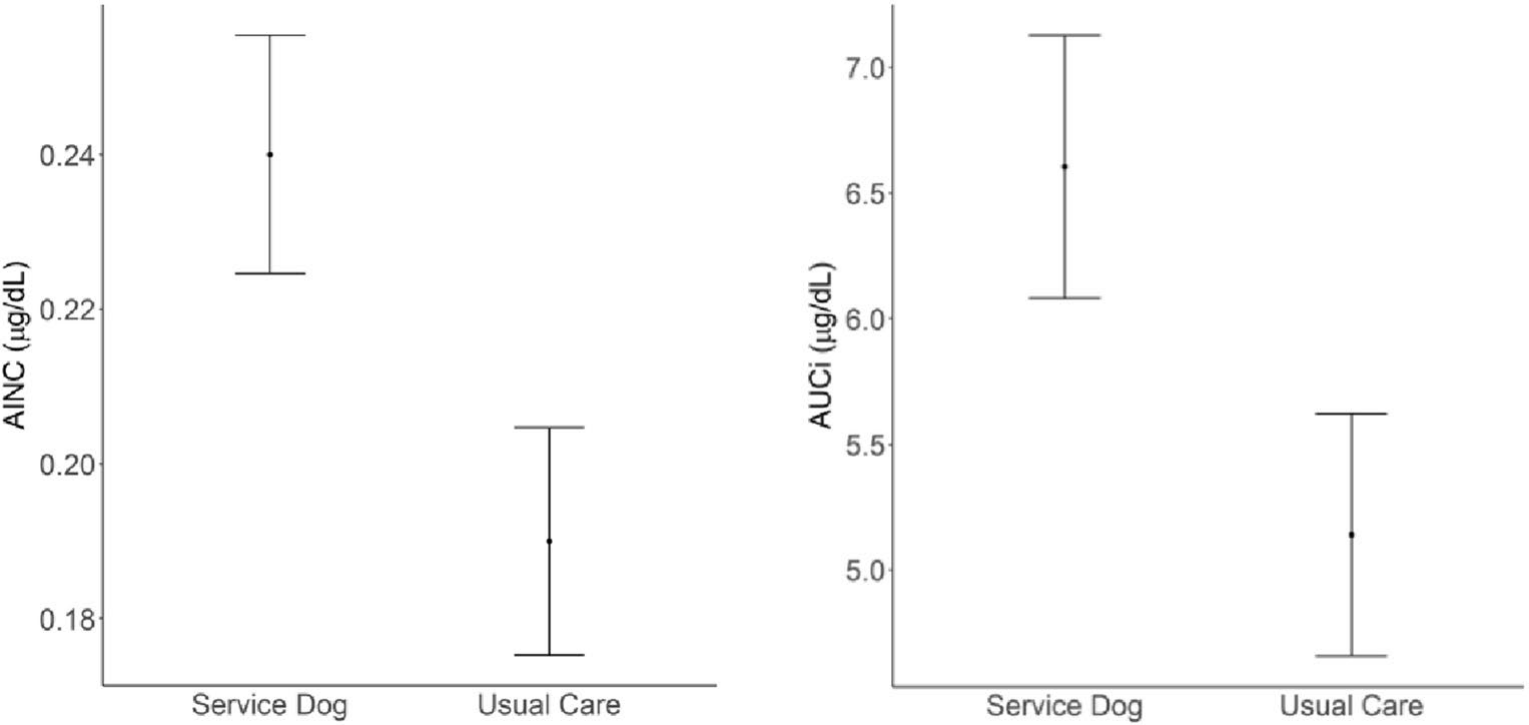
Mean Predicted CAR (AINC) and AUCi by group for veterans. *Note* Covariate adjusted means of CAR and AUCi controlling for baseline values, PANAS total score, VR-12 Physical Score as a measure of physical health, panic attack indicator, number of panic attacks, and pre-awakening activity. Bars represent standard error. *CAR (AINC) cortisol awakening response in (μg/dL), *AUCi area under the curve with respect to increase.

A total of 245 individuals participated in saliva sampling, including 161 veterans (*n* = 88 with a service dog, *n* = 73 receiving usual care) and 84 partners (*n* = 46 with a service dog,* n* = 38 receiving usual care; Table 1). Across both veterans and partners at baseline and 3 months follow up, a total of 3951 total samples were collected, representing 755 days of collection. Veterans collected 2613 salivary samples out of a possible 2898 samples planned (90%, three samples per day, over 3 days, at both timepoints). Samples represent collection from approximately 452 days across baseline and 3 months follow up. Partners collected 1338 salivary samples out of a possible 1512 samples planned (88%) and samples represent collection from 303 days across baseline and 3 months follow up. A total of 28.2% of veteran samples and 20.4% of partner samples were removed following the protocol for non-adherence. Results from the MMRM indicate that veterans with a service dog had a significantly higher CAR in comparison to the usual care group (Table 2). Both the area under the curve with respect to increase (AUCi, β = 1.46, SE = 0.73, *p* = 0.046) and the absolute increase (AINC, β = 0.05, SE = 0.02, *p* = 0.035) were significantly higher at follow up when controlling for baseline values (Fig. 1). PTSD Checklist (PCL-5) severity scores were not significantly correlated (< 0.1) with the AUCi or AINC. Results from the MMRM indicate that although the direction was positive (service dog group was higher), there was no significant difference in the CAR between partners of veterans with service dogs and partners of veterans without service dogs in terms of area under the curve with respect to increase (AUCi, β = 1.36, SE = 1.18, *p* = 0.255) or absolute increase (AINC, β = 0.04, SE = 0.04, *p* = 0.314).

**Table 1:** Demographics of participants across groups at Baseline.

	Veterans				Partners			
	Waitlist (*n* = 73)	Service dog (*n* = 88)	*t* or χ^2^	*p*	Waitlist (*n* = 38)	Service dog (*n* = 46)	*t* or χ^2^	*p*
Age, M (SD)	38.2 (8.6)	37.7 (8.5)	− 0.35	0.73	37.1 (8.8)	36.2 (7.6)	− 0.52	0.6
Gender, n (%)			0.64	0.42			-	1.0
Female	22 (30%)	20 (23%)			33 (87%)	41 (89%)		
Male	51 (70%)	66 (77%)			5 (13%)	5 (11%)		
BIPOC, n (%)	29 (40%)	29 (34%)	0.4	0.53	14 (37%)	13 (29%)	0.39	0.53
Socioeconomic status, n (%)			–	0.08			–	0.07
Comfortable	35 (49%)	26 (31%)			21 (55%)	13 (28%)		
Just enough to make ends meet	32 (44%)	51 (60%)			14 (37%)	27 (59%)		
Not enough to make ends meet	5 (6.9%)	8 (9.4%)			3 (7.9%)	6 (13%)		
Employed, n (%)	25 (35%)	29 (34%)	0.02	0.89	27 (71%)	28 (61%)	0.47	0.49
Education, n (%)			–	0.44			–	0.6
Some high school	0	0			0 (0%)	1 (2.2%)		
High school/GED	4 (5.6%)	7 (8.2%)			9 (24%)	6 (13%)		
Some college	22 (31%)	32 (38%)			11 (29%)	16 (35%)		
2 years degree	11 (15%)	17 (20%)			5 (13%)	6 (13%)		
4 years degree	19 (26%)	18 (21%)			8 (21%)	13 (28%)		
Post-graduate degree	16 (22%)	11 (13%)			5 (13%)	4 (8.7%)		
Relationship status, n (%)			–	0.09			–	0.44
Single (never married)	6 (8.2%)	14 (16%)			–	–		
Living with partner	3 (4.1%)	4 (4.7%)			2 (5.3%)	5 (11%)		
Married	53 (73%)	46 (53%)			36 (95%)	40 (87%)		
Divorced	9 (12%)	13 (15%)			0 (0%)	1 (2.2%)		
Separated	2 (2.7%)	9 (10%)			–	–		
Has pet(s), n (%)	47 (65%)	50 (59%)	0.44	0.51	28 (74%)	34 (74%)	0.01	0.92
Military branch, n (%)			–	0.09			–	–
Air force	5 (8.8%)	5 (6.5%)			–	–		
Army	31 (54%)	45 (58%)			–	–		
Navy	12 (21%)	5 (6.5%)			–	–		
Marine corps	7 (12%)	16 (21%)			–	–		
Coast guard	1 (1.8%)	1 (1.3%)			–	–		
National guard	1 (1.8%)	5 (6.5%)			–	–		
Current PTSD treatment, n (%)	62 (87%)	75 (90%)	0.12	0.73	1 (33%)	2 (22%)	–	1.00
Number of PTSD treatments in the past 3 months, M (SD)	9.1 (9.6)	10.3 (11.3)	0.69	0.49	–	–	–	–
PTSD severity score, M (SD)	56.3 (14.2)	57.0 (11.0)	0.36	0.72	16.7 (15.3)	18.8 (16.3)	0.61	0.55
Veteran’s rand physical composite score, M (SD)	38.8 (11.7)	40.2 (9.4)	0.83	0.41	–	–	–	–
TBI, n (%)	34 (47%)	37 (44%)	0.09	0.76	–	–	–	–
BMI, M (SD)	31.2 (6.8)	31.6 (6.3)	0.34	0.73	28.2 (4.7)	30.2 (7.4)	1.5	0.14
Smokes, n (%)	18 (25%)	30 (36%)	1.62	0.2	12 (32%)	6 (13%)	2.86	0.09
PROMIS alcohol, M (SD)	43.8 (7.3)	44.8 (8.6)	0.78	0.44	42.6 (5.9)	42.4 (5.6)	− 0.16	0.87
Steroid medication, n (%)	6 (8.6%)	1 (1.2%)	–	0.05	0 (0%)	0 (0%)	–	–
Pregnant, n (%)	0 (0%)	1 (5.0%)	–	0.48	1 (3.1%)	1 (5.9%)	–	1.00
Average sleep efficiency over baseline M (SD)	81.0 (10.1)	79.7 (10.1)	− 0.77	0.44	86.1 (4.9)	84.8 (5.8)	− 1.02	0.31
Average affect score over baseline M (SD)	− 2.1 (7.7)	− 5.9 (7.6)	− 3.11	0	7.3 (6.7)	6.9 (8.7)	− 0.26	0.80
Average s1 value over baseline, Mean (SD)	0.3 (0.1)	0.3 (0.1)	0.95	0.34	0.34 (0.5)	0.33 (0.2)	− 0.18	0.86

Statistical Tests performed: t-test (continuous), chi-squared test (binary categorical), Fisher’s exact test (non-binary categorical).

*BIPOC* black, indigenous, person of color, *TBI* deployment-related traumatic brain injury, *BMI* body mass index, *PROMIS* patient-reported outcomes measurement information system, *S1* cortisol sample taken upon awakening.

– indicates that no data was available.

**Table 2:** Veteran mixed model repeated measures analysis summary.

	AUCi			CAR (AINC)		
Variable	Estimate	SE	*p* value	Estimate	SE	*p* value
Intercept	2.593	1.816	0.154	0.117	0.053	0.026
Service dog	1.459	0.731	0.046*	0.045	0.021	0.035*
Baseline value	0.174	0.087	0.047*	0.141	0.079	0.077
Affect	− 0.104	0.037	0.005**	− 0.003	0.001	0.006**
Physical health	0.036	0.036	0.307	0.001	0.001	0.281
Binary panic attack	1.647	1.565	0.293	0.053	0.047	0.263
# of panic attacks	− 0.951	0.680	0.162	− 0.025	0.021	0.236
Pre-awakening activity	− 0.003	0.036	0.932	− 0.000	0.001	0.881

The usual care group is the reference group.

*AUCi* area under the curve with respect to increase, *CAR (AINC)* absolute increase of cortisol.

*p < 0.05, **p < 0.001.

## Discussion

The purpose of the current study was to empirically identify the impact that service dogs may have on the stress physiology of veterans and veteran partners through analysis of the cortisol awakening response (CAR). Results suggest that veterans with service dogs may have a significantly higher CAR in comparison to veterans receiving usual care alone. Given that individuals with PTSD may have dysregulation of the HPA axis, and subsequently dysregulation of the cortisol awakening response, results suggest that a service dog, as a complementary intervention, may offer a unique mechanism for regulation. These results are aligned with previous findings^9^ suggesting that veterans with a service dog have higher CAR than veterans on a waitlist for a service dog. Findings also suggest that affect may be minimally related to CAR. Though this finding could be considered counterintuitive to expectations, the size of the effect was negligible (− 0.104 and − 0.003 respectively), suggesting more targeted research is required to draw substantive conclusions, especially given the broader mixed findings in this domain^58,59^.Results from the veteran partners were not significant, indicating that the service dog may not influence partners through the same mechanism as veterans. This is not surprising as the role of the service dog is to offer individualized support to the veterans, not the partners.

Findings add information to previous studies that suggest a potential physiological mechanism for service dogs for veterans with PTSD. Previous studies have hypothesized that Attachment Theory and the role of a service dog as a secure base may help to lower stress levels by mitigating arousal, a common symptom associated with PTSD. It is possible that the severity of PTSD, and in particular the symptom of hyperarousal, plays an important role in this mechanism, potentially explaining differences in findings across studies^9,10^. Most of the current literature regarding the impact of service dogs on veterans focuses on psychological and social outcomes only. The addition of the biological findings in this study allow use of the biopsychosocial model of human health and wellbeing^11^. Understanding the interaction and outcomes of the three components is critical in identifying ways to individualize the intervention by highlighting specific mechanisms by which certain outcomes may occur.

Findings suggest that this potential mechanism is not found in military partners. The capacity by which partners interact with the service dogs is different than the interaction of the dog with the veterans. Service dogs are matched with veterans as an individualized complementary intervention for their PTSD. The service dogs are trained to do tasks that mitigate PTSD of the veterans, not to mitigate any specific need for the partners. Previous literature emerging from this same population of veteran partners suggests that partners may be influenced by the service dogs both positively in terms of higher positive emotions (e.g., calmness and confidence) and negatively in terms of lower caregiver satisfaction and higher caregiver burden^13,14^. Previous findings also suggest that there was no influence of service dogs on the anxiety and depression levels of partners^14^. Though previous literature suggests a potential indirect influence of the service dog on the partners, findings from this study suggest that the mechanism by which this influence is occurring may be different than what we are seeing with the veterans and not related to arousal dysregulation and the HPA axis.

### Limitations and future research

Though this study strengthens our understanding of the influence of a service dog on the CAR, there are multiple limitations to consider. First, capturing additional covariates in the model may provide more specific insights. Though the model accounted for the majority of the recommended covariates in the expert guidelines^44^, future studies should capture a broader understanding of medication use (beyond the use of steroids). The severity and location of any traumatic brain injuries (TBI) should also be included, beyond a binary variable that only focuses on deployment related TBI. Additionally, future studies should ask questions regarding female participants’ menstrual cycle and breastfeeding status. Second, given the sensitivity of the CAR, adherence can be difficult. Though non-adherence was accounted for in multiple ways within the study design (e.g., text-message time stamps, actigraphy, morning sleep diaries), there was still unaccounted variance potentially because of the in-home study environment. Future studies should consider developing additional strategies regarding improving compliance, potentially collecting samples in a more controlled laboratory environment. Third, given the complexity of hyperarousal, additional physiological measures (e.g., heartrate) should be considered in tandem with CAR to more fully capture the concept of arousal in this context. Lastly, these findings capture the experience of individuals from one service dog provider. It is unknown whether these findings are similar across providers. Future studies could be designed and powered to look at the individual differences of CAR more specifically, perhaps focusing on differences between treatment non-responders and responders (as evidenced by PTSD changes and/or CAR status) or employing a latent state trait analysis for further understanding of the role of these individual differences^60^.

## Conclusion

Results from this longitudinal clinical trial suggest that veterans with service dogs have a higher CAR than veterans in the usual care control group. When controlling for baseline values, both the area under the curve with respect to increase and the absolute increase were significantly higher at follow up for veterans with a service dog. These findings replicate a previous cross-sectional study with a similar population and suggest that service dogs may influence hyperarousal of veterans through modulation of the HPA axis^9^. Results for partners were null, suggesting that this potential mechanism is not found in military partners. Taken together, these findings advance the understanding of biological processes and potential mechanisms underlying the impact of service dogs for military veterans with PTSD.

## Data Availability

The data that support the findings of this study are available on request from the corresponding author, MO. The data are not publicly available due to their containing information that could compromise the privacy of research participants.
